# Molecular Requirements for Self-Interaction of the Respiratory Syncytial Virus Matrix Protein in Living Mammalian Cells

**DOI:** 10.3390/v10030109

**Published:** 2018-03-03

**Authors:** Marta Trevisan, Veronica Di Antonio, Annalisa Radeghieri, Giorgio Palù, Reena Ghildyal, Gualtiero Alvisi

**Affiliations:** 1 Department of Molecular Medicine, University of Padua, Padua 35121, Italy; marta.trevisan@unipd.it (M.T.); veronicadiantonio@libero.it (V.D.A.); giorgio.palu@unipd.it (G.P.); 2 Department of Molecular and Translational Medicine, University of Brescia, Brescia 25123, Italy; annalisa.radeghieri@unibs.it; 3 Centre for Research in Therapeutic Solutions, Faculty of Science and Technology, University of Canberra, Canberra 2617, Australia

**Keywords:** RSV M protein, virus assembly, Bioluminescence resonance energy transfer (BRET), confocal microscopy

## Abstract

Respiratory syncytial virus (RSV) is an important human pathogen, which infects respiratory tract epithelial cells causing bronchiolitis and pneumonia in children and the elderly. Recent studies have linked RSV matrix (M) ability to self-interaction and viral budding. However, RSV M has been crystalized both as a monomer and a dimer, and no formal proof exists to date that it forms dimers in cells. Here, by using a combination of confocal laser scanning microscopy and bioluminescent resonant energy transfer applied to differently tagged deletion mutants of RSV M, we show that the protein can self-interact in living mammalian cells and that both the N and C-terminus of the protein are strictly required for the process, consistent with the reported dimeric crystal structure.

## 1. Introduction

Respiratory syncytial virus (RSV) is the major cause of lower respiratory tract disease in infants and young children [1,2,3], responsible for one-third of deaths resulting from acute lower respiratory infection in the first year of life [4,5,6]. RSV also causes severe respiratory tract disease in immunosuppressed and older adults, leading to substantial annual mortality [7]. There are no vaccines or antiviral drugs that effectively target RSV despite decades of research [8]. Deeper understanding of the molecular mechanisms that underlie RSV assembly could pave the way to the identification of new vaccine/antiviral targets.

RSV is an enveloped virus with a non-segmented negative sense RNA genome and belongs to the *Orthopneumovirus* genus of the *Pneumoviridae* family [9]. The RSV genome is tightly encapsidated within the nucleocapsid, which is composed of nucleocapsid protein N, the RNA polymerase L and its cofactor phosphoprotein P, as well as the M2-1 protein. External to the nucleocapsid is a layer of matrix (M) protein which acts as a bridge between the nucleocapsid and the lipid bilayer envelope. Embedded in the envelope are the fusion (F), large (G) and small hydrophobic (SH) glycoproteins. M2-2, NS1 and NS2 proteins are not found in the virion in any significant amount but have important roles in the RSV replication cycle [10,11,12,13,14,15].

M protein is a major structural protein of RSV, playing a central role in virus assembly and in retaining the intact virion [16]. M interacts with the envelope glycoproteins [17,18,19,20], with the nucleocapsids [21,22,23,24] and with the host membrane [19,25,26] to facilitate assembly. M has been postulated to bring the nucleocapsids and envelope glycoproteins together through its ability to oligomerise [27,28]. This is an essential assembly step to ensure production of infectious virus. Previous studies have shown that M readily forms homodimers and self-aggregates in vitro [19,28,29]. Despite the harsh ionic conditions required to isolate M in its monomeric form [19], M was at first crystallized as a monomer [26] and later shown to form dimers [27], similarly to matrix proteins from other *Mononegavirales*, including other *Penumoviridae* members such as human Metapneumovirus [27,30,31,32,33]. Based on the in vitro data and on observations of mutant M in the context of infected and transfected cells, it was postulated that M can form oligomers in cells, however this had not been shown experimentally. Mutations targeting the dimerization interface affected the ability to form virus-like particles in a co-transfection system, but this did not correlate with loss of dimerization as assessed by size exclusion chromatography [27], raising the possibility that the observed phenotype was due to misfolding or aggregation of M.

In this study, we have used confocal laser scanning microscopy (CLSM) and bioluminescent resonant energy transfer (BRET) in cells transfected to express differently tagged M, to show that M can self-interact in live cells and that both N and C termini are required for this interaction. Our findings confirm and extend previous in vitro data on M oligomerization. Importantly, our studies validate the published structure of the M dimer that predicts a direct interaction between N and C termini of two monomers to form the dimer [27].

## 2. Materials and Methods

### 2.1. Plasmid Construction

Mammalian expression plasmids were generated using the Gateway^TM^ technology (Invitrogen, Carlsbad, CA, USA). Entry clones pDNR207-M (1–256), pDNR207-M (1–200), pDNR207-M (110–183) and pDNR207-M (183–256) were generated via BP recombination reactions between PCR products with *att*B sites flanking the appropriate coding sequences and plasmid pDNR207 (Life Technologies, Carlsbad, CA, USA, as described previously [34]), using the full-length codon optimized M gene cloned into pCDNA3.1 [27] as a template.

Entry clones were used to generate C-terminal yellow fluorescent protein (YFP), cyan fluorescent protein (CFP) and Renilla luciferase (RLuc) fusion Mammalian expression vectors following LR recombination reactions with the pDESTnYFP, pDESTnCFP and pDESTnRLuc [35] Gateway compatible vectors, as described in [36]. All vectors were confirmed by sequencing.

### 2.2. Cell Culture and Transfections

Human embryonic kidney (HEK)293-A and HEK293-T cells were maintained in Dulbecco’s modified Eagle’s medium (DMEM) supplemented with 10% (*v*/*v*) fetal bovine serum (FBS), 50 U/mL penicillin, 50 U/mL streptomycin and 2 mM l-glutamine as described in [37]. For imaging experiments, cells were trypsinized and 2.5 × 10^4^ HEK293-A cells were seeded onto polylysinated 12 mm glass coverslips in 24-well plates 1 day before transfection [38]. Each well was transfected with a total of 250 ng of plasmid DNA and 1 μL of lipofectamine 2000 (Thermofisher, Waltham, MA, USA).

For BRET experiments, cells were trypsinized and 1 × 10^5^ HEK293-T cells were seeded onto 24-well plates 1 day before transfection [39]. Each well was transfected with a total of 500 ng of plasmid DNA and 2 μL of lipofectamine 2000 (Thermofisher). BRET saturation experiments were performed transfecting cells with 0.5 ng of RLuc-M (1–256) and increasing amounts (0–450 ng) of YFP-M (1–256). Total DNA amount was normalized to 500 ng total with plasmid pCDNA3.1 (Thermofisher, Waltham, MA, USA). Importantly, no signs of cell toxicity were observed upon transfection of all M expression plasmids.

### 2.3. Microscopy/CLSM/Image Analysis

Subcellular localization of fluorescently tagged fusion proteins was visualized 24 h and 48 h after transfection using an inverted epi-fluorescent microscope (Leica, Wetzlar, Germany) equipped with a 40× objective, essentially as described previously [40]. 48 h after transfection, cells were fixed with 4% paraformaldehyde 15 min at room temperature (RT), before being mounted onto glass coverslips with FluoromountG (Southern Biotech, Birmingham, AL, USA). When required nuclei where counterstained with DRAQ5 (Life Technologies, Carlsbad, CA, USA, 1:1000). Samples were processed by confocal laser scanning microscopy (CLSM) using a Leica TCT-SP2 system, equipped with a Planapo fluor 63× oil immersion objective (Leica). The Fn/c values were determined using the NIH ImageJ 1.62 public domain software, from single cell measurements for each of the nuclear (Fn) and cytoplasmic (Fc) fluorescence, subsequent to the subtraction of fluorescence due to autofluorescence/background as described previously [41]. Co-localization analysis was performed using the coloc2 plugin. Data were plotted and analyzed using Prism 6 (GraphPad) software (La Jolla, CA, USA).

### 2.4. Bioluminescence Resonance Energy Transfer (BRET) Assays

BRET experiments were performed as described in [35]. Briefly, 293T cells were transfected in 24-well plates with appropriate amounts of BRET donor expressing plasmids. For each construct, the donor (RLuc) expressing plasmid was transfected either in the absence or in the presence of the relative acceptor (YFP) expressing plasmid to allow calculation of background BRET signal. 48 h post transfection, culture media was removed from wells and cells were very gently washed with 1 mL of PBS, before being resuspended with 290 µL of fresh PBS. Cells were further resuspended and 90 µL of mixture were transferred to a black bottomed 96-well plate (Costar^®^, Washington, DC, USA, product number 3916) well in triplicate, and signals acquired using a spectrometer compatible with BRET measurements (VICTOR X2 Multilabel Plate Reader, PerkinElmer, Waltham, MA, USA). Fluorescent signal (YFPnet) relative to YFP fluorescent emission were acquired using a fluorimetric excitation filter (band pass 485 ± 14 nm) and a fluorimetric emission filter (band pass 535 ± 25 nm). Luminometric readings were performed at 5’, 15’, 30’, 45’ and 60’ after addition of the substrate (native Coelenterazine or Coelenterazine-h, depending on the assay, 5 µM PJK, Kleinblittersdorf, Germany). Data were acquired for 1 s/well, using a luminometric 535 ± 25 nm emission filter (YFP signal) and a luminometric 460 ± 25 nm emission filter (RLuc signal). Before reading, the plate was shaken for 1 s at normal speed and with double orbit. After background subtraction using values relative to mock transfected cells, the data obtained were used to calculate the BRET signal, defined as the ratio between the YFP and RLuc signals calculated for a specific BRET pair, according to the formula:

$$\text{BRET signal}=\frac{\text{YFP signal}}{\text{RLuc signal}}$$

Similarly, the BRET ratio, defined as the difference between the BRET value relative to a BRET pair and the BRET value relative to the BRET donor alone, was calculated according to the formula:

$$\text{BRET ratio}=\frac{\text{YFP signal}}{\text{RLuc signal}}\text{BRET pair}-\frac{\text{YFP signal}}{\text{RLuc signal}}\text{BRET donor}$$

BRET saturation curves were then calculated using the GraphPad Prism software by plotting each individual BRET ratio value to the YFPnet/RLuc signal, and interpolating such values using the one-site binding hyperbola function of GraphPad Prism. Specific BRET pairs generate logarithmic shaped curves and reach a plateau. This allowed calculation of BRETmax (Bmax) and BRET_50_ (B_50_) values, indicative of maximum energy transfer and relative affinity of the BRET pair tested. 

### 2.5. Visualization of RSV M Crystal Structures

PDB file 42V3 was downloaded from the protein data bank website and Molecular graphics and analyses were performed with the UCSF Chimera package [42]. Chimera is developed by the Resource for Biocomputing, Visualization, and Informatics at the University of California, San Francisco (supported by NIGMS P41-GM103311).

## 3. Results

### 3.1. Deletion of N- and C-Terminal Portions of RSV M Affects Protein Subcellular Localization

**Figure 1:**
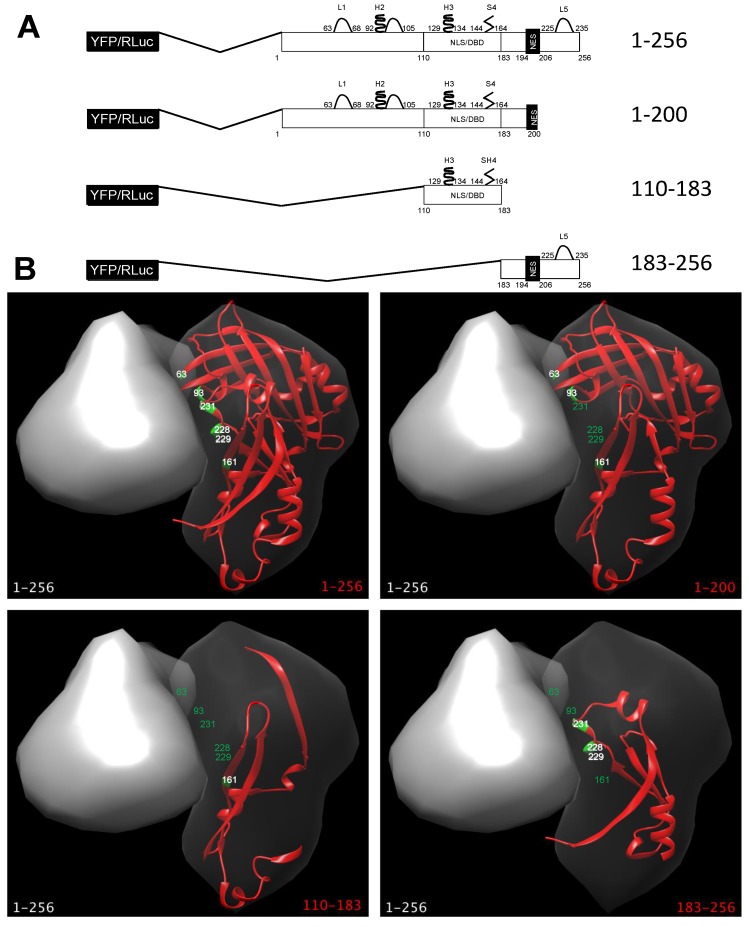
M deletion mutants used in this study (**A**) Schematic representation of R espiratory syncytial virus (RSV) matrix (M) deletion mutants used in this study as fused to either YFP, RLuc and cyan fluorescent protein (CFP). Elements involved in dimerization are shown, based on the nomenclature used in [26]*.* L1, loop 1 (aas 63–88); H2, helix 2 and downstream loop (aas 92–105); H3, helix 3 (aas 129–134); S4, sheet 4 (aas 144–163); L5, loop 5 (225–235); NLS/DBD, nuclear localization sequence/DNA binding domain (residues 110–183); NES, nuclear export sequence (aas 194–206); YFP, yellow fluorescent protein; RLuc, Renilla luciferase; (**B**) The recently solved M dimeric structure (PDB code 4V23) was used to highlight the key aas involved in M dimerization and their position relative to each deletion mutant tested in this study, using software Chimera as described in the Materials and Methods section. The surface of a full-length M monomer (1–256) is shown as a grey structure in combination with the M versions used in this study, shown as red ribbons. Key residues involved in dimerization in the latter subunit are shown in green. Residue labels are shown either in white or green, depending on their presence or absence in the corresponding deletion mutant, respectively.

We aimed to investigate whether M exists as a dimer/oligomer within cells, and which protein domains are involved in dimerization. We initially analyzed the subcellular localization of several RSV M deletion mutants as expressed in Mammalian cells when C-terminally fused to YFP and CFP. Such fusions included: full-length (FL) M (1–256); M (1–200), lacking a C-terminal loop (L5) involved in formation of dimeric structures by interacting with N-terminal helix H2 and loop L2, as well as the most extreme C-terminal residues of M nuclear export signal (NES): aas 194–206); M (110–183), containing only the nuclear localization signal (NLS) and DNA binding domain (DBD) (NLS/DBD, residues 110–183), along with a α-sheet (S4) involved in protein dimerization by interacting with loop L5, but lacking all other functional elements; M (183–256), retaining only the C-terminal NES and loop L5 (Figure 1).

**Figure 2:**
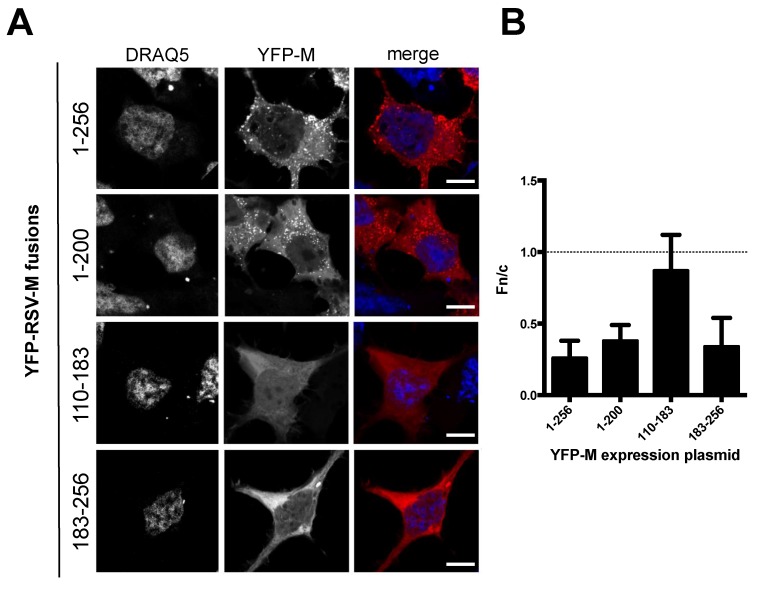
Deletion of N- and C-terminal portions of RSV M affects protein subcellular localization. (**A**) HEK293 A cells were transfected to transiently express the indicated YFP-M fusions. 24 h post transfection cell nuclei were stained with DRAQ5, and samples processed for confocal laser scanning microscopy (CSLM) analysis. Representative images relative to cell nuclei (DRAQ5) and M fusions (YFP-M) are shown on the left and middle panels, respectively. Merged images of the two channels are shown in the right panels. Scale bars represent 20 μM. (**B**) Digital images such as those shown in (**A**) were quantitatively analyzed using software ImageJ to calculate the Fn/c ratio relative to each fusion protein, as described in Material and Method section. The mean ± SD relative to at least 75 cells from 2 independent experiments is shown. The dotted line represents Fn/c of 1, corresponding to an even distribution between the nucleus and the cytoplasm.

When expressed individually, the proteins differentially distributed within the cells (Figure 2A,B). YFP-M (1–256) localized mainly in the cytosol, with only very faint nuclear staining (Fn/c = 0.26 ± 0.12), consistent with the presence of a strong, chromosomal maintenance 1 (CRM-1) dependent NES, with a punctate pattern reminiscent of ER/Golgi trafficking. The subcellular localization of YFP-M (1–200) was similar to that of YFP-M (1–256), with the exception that a higher fraction of the protein localized to the nucleus (Fn/c = 0.38 ± 0.12), and with the fact that the punctate staining was evident in a lower percentage of cells, most likely due to lower expression levels. The difference in the nuclear distribution of YFP-M (1–200) compared to FL M is probably due to the presence of the NLS and the partial loss of the NES (see Figure 1A). Removal of M N-terminal domain resulted in YFP-M (183–256) localizing mainly to the cytosol (Fn/c = 0.34 ± 0.12), mainly with a diffuse pattern, consistently with the presence of a functional NES. On the other hand, YFP-M (110–183), accumulated to the nucleus to higher extent as compared to the other M fusions, in accordance with the complete deletion of M NESs (Fn/c = 0.9).

### 3.2. Deletion of N- and C-Terminal Portions of RSV M Affects M’s Ability to Colocalize with Full-Length Protein

**Figure 3:**
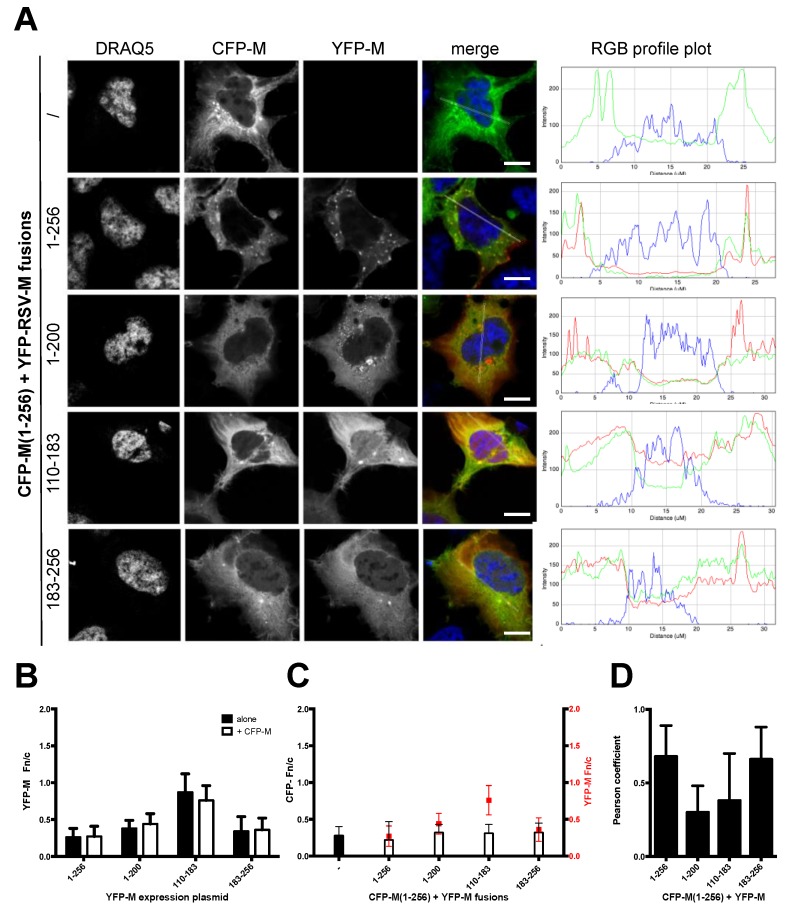
Deletion of N- and C-terminal portions of RSV M affects protein ability to colocalize with full-length protein. (**A**) HEK293 A cells were transfected to transiently express the CFP-M (1–256) fusion protein either in the absence or in the presence of the indicated YFP-M fusions. 24 h post transfection cell nuclei were stained with DRAQ5 and cells processed for CSLM analysis. Representative images relative to cell nuclei (DRAQ5), CFP (CFP-M) and YFP-RSV M fusions (YFP-M) are shown, along with merged images of the three channels (merge). A RGB profile plot relative to the section highlighted in the merge channel is shown on the right panels; (**B**) Digital images such as those shown in (**A**) were quantitatively analyzed using software ImageJ to calculate the Fn/c ratio relative to each of the indicated YFP fusion protein, either expressed alone (black columns) or in the presence of CFP-M (1–256) (white columns). *n* ≥ 63 from at least two independent experiments; (**C**) Digital images such as those shown in (**A**) were quantitatively analyzed using software ImageJ to calculate the Fn/c ratio relative to CFP-M (1–256), either expressed alone (black columns) or in the presence of (white columns) of each of the indicated YFP fusion protein. Red dots indicate the Fn/c ratio relative to the indicated YFP-M fusions. *n* ≥ 63 from at least two independent experiments; (**D**) Images such as those shown in (**A**) were used to calculate the Pearson’s coefficient relative to each protein pair. *n* ≥ 19 from at least two independent experiments. Scale bars represent 20 μM.

We decided to investigate whether the elements involved in dimer formation in vitro [27] were required for M self-interaction in cells. To this end, we expressed CFP-M (1–256) in the presence or in the absence of the M deletion mutants as fused to YFP and investigated the ability of each fusion protein to co-localize, as well as to reciprocally affect each other’s subcellular localization. As expected, expression of CFP-M (1–256) resulted in a mainly cytosolic protein, occasionally showing a punctate pattern within the cytosol (Fn/c = 0.28 ± 12). Our data indicate that co-expression between CFP-M (1–256) and all YFP-M mutants tested in this study did not affect their reciprocal subcellular localization, as compared to when expressed individually (see Figure 3A–C).

Furthermore, while clear co-localization between CFP-M (1–256) and YFP-M (1–256) was observed in cytosolic dots (see Figure 3A,D; Pearson 0.68), such phenomena were not observed between CFP-M (1–256) and neither YFP-M (1–200) nor YFP-M (110–183), suggesting that the M deletion mutants tested are not capable of interacting with M (1–256) and affecting its subcellular localization. This hypothesis is also supported by the evidence that these deletions affected the Pearson colocalization coefficient with CFP-M (1–256), although to different extents (Figure 3D). Limited colocalization of YFP-M (183–256) with CFP-M (1–256) was observed in cytosolic dots (Figure 3A, compare the localization of CFP-M and YFP-M in cytosolic dots, bottom images) with a Pearson’s coefficient comparable to that between CFP-M (1–256) and YFP-M (1–256) (Figure 3D).

### 3.3. RSV M Can Self-Interact in Living Cells

**Figure 4:**
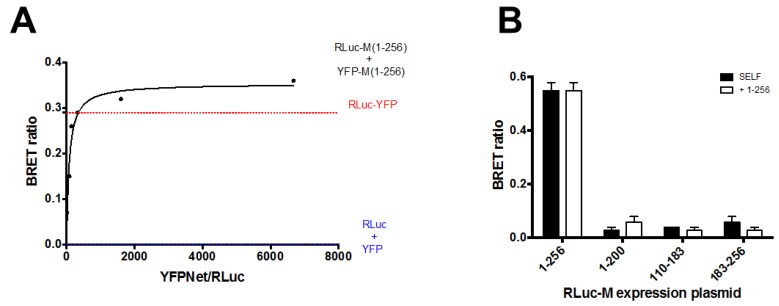
RSV M can self-interact in live Mammalian cells, depending on its N and C-terminal domains. (**A**) HEK293T cells were transfected to transiently express the RLuc-M (1–256) plasmid (0.5 ng) either in the absence or in the presence of increasing amounts the YFP-M (1–256) plasmid (range 0–450 ng). Alternatively, cells were transfected with the positive control plasmid RLuc-YFP (225 ng) or with plasmid RLuc-M (1–256) and pCMVFLAG-X-YFP as negative controls. 48 h later cells were processed for BRET measurements as described in the Materials and Methods section. The BRET ratio relative to the RLuc-M (1–256) and the YFP-M (1–256) BRET pair was plotted against the normalized YFPNet/RLuc ratio, and data used to calculate the Bmax and B50. The red dotted line indicates the BRET ratio obtained for the RLuc-YFP control protein, and the blue dotted line indicates the BRET ratio obtained for the RLuc-M (1–256) protein expressed in the presence of YFP alone. Representative data from four independent experiments are shown; (**B**) HEK293T cells were transfected to transiently express the RLuc-M expression plasmids either alone or in the presence of the indicated YFP-M expression plasmids. 48 h later cells were processed for BRET measurements as described in the Materials and Methods section. The BRET ratio relative to each condition is shown. Representative data from two independent experiments are shown.

Our results suggest that deletion of M C- or N-terminal domain affects its ability to self-interact in a cellular context. However, they do not prove that the full-length protein is able to self-interact or that the C-terminal of the protein is able to interact with the full-length protein. Indeed, the co-localization observed between CFP and YFP tagged versions of full-length M may simply reflect the fact that tagging M with such spontaneously fluorescent proteins does not affect its subcellular localization, so that both CFP- and YFP-tagged version of M localize in the same area of the cell. We directly addressed this issue by bioluminescent energy resonant energy transfer (BRET) assays. To this end, BRET saturation experiments were performed by transfecting HEK293-T with a fixed amount of BRET DONOR plasmid RLuc-M (1–256; 0.5 ng) in the presence of increasing amounts of BRET ACCEPTOR plasmid YFP-M (1:256; 0–450 ng). As a positive control and a reference for data normalization, a fusion protein between RLuc and YFP (RLuc-YFP) was also expressed, and RLuc and YFP were individually co-expressed as a negative control (Figure 4A).

At 48 h post transfection, YFP fluorescent and BRET signals were acquired in living cells, and BRET ratios calculated, as described in the Materials and Methods section. Our results indicate that the RLuc-YFP fusion generated a BRET ratio of 0.34 ± 0.02 (Figure 4A), while the RLuc and YFP protein, generated a BRET ratio of 0. Importantly, co-expression of RLuc-M (1–256) and YFP-M (1–256) generated a BRET ratio which rapidly increased with the ratio between YFP-M (1–256) and RLuc-M (1–256) expression levels (Figure 4A), and which quickly reached saturation. Data fitting allowed us to calculate the Bmax, corresponding to the maximal BRET ratio obtainable for the BRET pair (0.43 ± 0.04) and the B_50_, corresponding to the ratio between YFP-M (1–256) and RLuc-M (1–256) sufficient to generate a BRET ratio corresponding to half of the Bmax (48.4 ± 13.7). Overall, our results indicate that RLuc-M and YFP-M can interact with high affinity in live mammalian cells.

### 3.4. Deletion of N- and C-Terminal Portions of RSV M Affects Protein Ability to Form Dimers in Living Cells

We next used BRET assays to evaluate the ability of M deletion mutants to self-interact and to form complexes with the full-length protein. To this end, HEK293-T cells were transfected with a series of plasmids encoding the above described M deletion mutants fused to RLuc, either in the absence or in the presence of YFP-M expressing plasmids. Each RLuc-M derivative was expressed in the presence of its YFP-tagged version, or in the presence of YFP-M (1–256). 48 h post transfection cells were processed for BRET assays to monitor protein self-interaction. As expected, we could calculate a strong BRET signal (0.55 ± 0.03) relative to the RLuc-M (1–256)/YFP-M (1–256) BRET pair (Figure 4B), consistent with the fact that full-length M is capable of self-interacting in living mammalian cells. However, very weak BRET signals were calculated for the RLuc-M (1–200)/YFP-M (1–200), RLuc-M (110–183)/YFP-M (110–183) and RLuc-M (183–256)/YFP-M (183–256) BRET pairs (0.03, 0.04, 0.06, respectively, see Figure 4B, black bars), indicating both N- and C-terminal domains of M are required for homodimerization. Similarly, weak BRET signals were generated when the RLuc deletion mutants were expressed in the presence of full-length YFP-M (1–256), indicating that N- and C-terminal deletion mutants of M are not capable of interacting with full-length M in living cells (Figure 4B, white bars).

## 4. Discussion

The data presented in the current study shows that the RSV M protein can self-interact when expressed in living mammalian cells. Our study confirms in cell culture the self-interaction of M, previously shown by a number of studies reporting its ability to form homodimers and higher order oligomers in vitro [19,28,29], highlighting the physiological relevance of the in vitro observations.

Our discovery that this interaction in a cellular context requires both the N and C terminal domains of the protein is consistent with a recently reported head to tail dimeric structure of M, whereby the N-terminal domain of one subunit interacts with the C-terminal domain of the other subunit [27], and is in contrast with initially resolved monomeric M structure [26].

When transiently expressed in mammalian cells as YFP-M fusions, full-length M and its deletion mutants localized to the expected cellular compartment, depending on the presence or the absence of M NES and NLS, thus confirming and validating our previous work defining the nuclear transport motifs of M (see Figure 2; [43,44]). Importantly, YFP-M (1–200), lacking two leucine residues belonging to M NES (residues 194–206), localized significantly more in the nucleus than FL YFP-M. As expected, YFP-M (110–183), lacking the NES but bearing the NLS was present equally within the nucleus and the cytoplasm as has been shown previously, while YFP-M (183–256) that has the NES but lacks the NLS, was cytoplasmic [43].

Subcellular localization and co-localization analysis upon co-expression of CFP-M (1–256) with YFP-M (1–256) or its deletion mutants suggested that CFP-M (1–256) does not likely interact with any of the deletion mutants, with the possible exception of YFP-M (183–256), as indicated by the drop in co-localization (see Figure 3D). Furthermore, the subcellular localization of CFP-M (1–256) and YFP-M (110–183) was not affected upon co-expression, with the former remaining mainly cytosolic and the latter equally distributing between the cytoplasm and the nucleus (see Figure 3A–C). In contrast, YFP-M (183–256) partially co-localized with CFP-M (1–256), thus implying potential interaction between the two proteins. However, the co-localization observed likely reflects their presence in the same location and not necessarily an interaction (see below). Interestingly, YFP-M (1–200) formed cytoplasmic inclusion bodies (IBs) that look very similar to those formed by the CFP-M (1–256), yet when the two proteins were co-expressed, they did not co-localize. Expression of YFP- and CFP- fused deletion mutants also suggested that M (1–200) and M (183–256) may be able to self-interact as they formed IBs when expressed in living cells.

BRET analysis in living cells clearly showed that full-length M is very effective in forming dimers in living cells (see Figure 4A). However, none of the deletion mutants analyzed in our study were able to self-interact (see Figure 4B). This suggests that the observed IBs are formed due to aggregation that may be brought about by misfolding of the proteins. However, massive misfolding of the M mutants tested in our study is unlikely, since similar deletion mutants (containing M aas 1–144, 114–256 and 1–110), still interact with viral nucleocapsids to similar levels as the full-length protein [22]. Furthermore, RSV M (110–183) has been shown to inhibit host cell transcription to similar levels as compared to the full-length protein (Ghildyal et al., unpublished observations [45]). Our data is consistent with the M dimer being formed by the head to tail interaction of the subunits. In addition, none of the deletion mutants were able to dimerize with the full-length M (see Figure 4). This finding is consistent with the structure of the M dimer, which has a very large interface [27].

The dimerization interface comprises residues 63 to 68, 92 to 105, 129 to 134, 144, 163, 225 to 235, while residue T205 likely modulates M oligomerization in a phosphorylation dependent manner [27]. Our data shows that all the residues are needed to form a stable dimer, consistent with previous work, which demonstrated that mutation of single residues in the context of the full-length protein has an observable effect on the filament formation and dimerization/oligomerization behavior of M [27]. Clearly, there are complex interactions with several residues in the dimerization interface with each having a specific role in stabilizing the structure. Since point mutations destabilizing RSV M self-interaction in vitro also negatively affected viral budding, it is reasonable to consider M dimerization as an attractive potential target for the development of antiviral agents. In this context, the BRET-based assay described here to monitor M self-interaction might provide a valuable tool for screening of compounds interfering with M self-interaction, or for the validation of hits identified by other methods [35,46,47,48].
